# Probiotic treatment induces intestinal regulatory dendritic and T cells, and counter-regulates Th2 responses and anaphylaxis in a mouse model of food allergy

**DOI:** 10.1186/2045-7022-1-S1-O30

**Published:** 2011-08-12

**Authors:** Elisa Schiavi, Bianca Barletta, Andrea Barone, Cinzia Butteroni, Silvia Corinti, Gabriella Di Felice

**Affiliations:** 1 Istituto Superiore di Sanità, Dept. Infectious, Parasitic and Immune-mediated Diseases, Rome, Italy

## 

The immunological mechanisms responsible for the anti-allergic effects of probiotic bacteria are still poorly defined. We tested the effect of a probiotic mixture (VSL#3) in in vitro, ex vivo and in vivo mouse systems. In vitro co-culture of naïve bone marrow(BM)-derived DC (BM-DC) with VSL#3 induced the up-regulation of maturation marker and IL-10 and IL-12 production. Ex vivo analysis of mesenteric lymph node(MLN)-derived DC (MLN-DC) from naïve mice receiving for three weeks VSL#3 by oral administration, indicated a different distribution and phenotype of DC within the MLN. In particular, VSL#3 treatment increased the frequency of plasmacytoid DC (pDC, B220+CD11clow), and upregulated the expression of maturation markers on conventional DC (cDC). Moreover, the frequency of IL-10-expressing cDC was increased. This finding was paralleled by the increase of CD4+CD25+ T cells and CD4+CD25- populations showing enhanced IL-10 production. Altogether, these results suggest that VSL#3 treatment can stimulate in the gut the activation of tolerogenic DC. Finally, we obtained in vivo preliminary data on therapeutic and preventive potential of VSL#3 in a mouse model of sensitization and anaphylaxis to peanut. Mice were orally sensitized and challenged with peanut extract to induce in vivo anaphylaxis. In the therapeutic experimental setting, animals received a three-weeks oral treatment with VSL#3 and were then re-challenged. In the preventive setting, oral probiotic treatment started one week before the beginning of the immunization schedule and continued until the challenge. In both approaches, VSL#3 was able to reduce anaphylaxis symptoms and IL-13 release in the jejunum of immunized mice upon post-treatment challenge. Furthermore, the therapeutic approach also induced allergen-specific IgA in the gut and TGF-β release. Then, the capacity of probiotics to induce protective immune responses linked to counter-regulation of Th2 responses might become an effective strategy in the treatment of type I allergy.

